# Valproic Acid Stimulates Release of Ca^2+^ from InsP_3_-Sensitive Ca^2+^ Stores

**DOI:** 10.3390/ijms27031176

**Published:** 2026-01-23

**Authors:** Ana Ruiz-Nuño, María F. Cano-Abad

**Affiliations:** 1 Departamento de Farmacología, Facultad de Medicina, Universidad Autónoma de Madrid, 28029 Madrid, Spain; anaruiznuno@yahoo.es; 2 Instituto de Investigación Biomédica, Hospital Universitario de la Princesa, 28006 Madrid, Spain

**Keywords:** valproic acid, InsP_3_ receptor-mediated Ca^2+^ release, endoplasmic reticulum Ca^2+^ release, intracellular Ca^2+^ signaling, cytosolic Ca^2+^ dynamics, ER Ca^2+^ homeostasis, aequorin, fura-2

## Abstract

Calcium (Ca^2+^)signaling dysfunction is a central contributor to neuronal hyperexcitability and seizure propagation in epilepsy, yet the intracellular mechanisms underlying the actions of valproic acid (VPA) remain incompletely understood. In this study, we investigated whether VPA modulates Ca^2+^ homeostasis at the level of the endoplasmic reticulum (ER) and how this action influences cytosolic Ca^2+^ dynamics associated with epileptiform activity. ER Ca^2+^ levels were directly measured using ER-targeted aequorin in HeLa and PC12 cells, while cytosolic Ca^2+^ signals were monitored by fura-2 fluorescence imaging in bovine chromaffin cells exposed to veratridine, a model of sustained sodium channel activation and Ca^2+^ oscillations. VPA induced a concentration-dependent release of Ca^2+^ from the ER, with an IC_50_ of approximately 17 µM. This effect was preserved in permeabilized cells and exhibited activation kinetics comparable to those elicited by inositol 1,4,5-trisphosphate (InsP_3_). Pharmacological inhibition of InsP_3_ receptors (InsP_3_Rs), but not ryanodine receptors or SERCA, abolished VPA-induced ER Ca^2+^ release, supporting a selective InsP_3_R-mediated mechanism. Functionally, VPA suppressed the repetitive cytosolic Ca^2+^ oscillations induced by veratridine, while simultaneously producing a sustained elevation of cytosolic Ca^2+^ originating from ER stores and facilitating depolarization-evoked catecholamine secretion. Together, these results support the conclusion that VPA induces InsP_3_R-mediated Ca^2+^ mobilization from the endoplasmic reticulum and identify ER Ca^2+^ release as a previously unrecognized intracellular mechanism contributing to its modulatory effects on Ca^2+^ signaling and excitability in epilepsy.

## 1. Introduction

Epilepsy is a chronic neurological disorder characterized by recurrent seizures arising from abnormal, synchronized neuronal discharges. The pathophysiology of epilepsy involves a complex interplay of excitatory and inhibitory mechanisms in the brain, where Ca^2+^ ions play a fundamental regulatory role. Intracellular Ca^2+^ fluctuations are essential for neuronal excitability, neurotransmitter release, gene expression, and synaptic plasticity processes such as long-term potentiation [1,2]. However, prolonged or excessive increases in intracellular Ca^2+^ concentration ([Ca^2+^]_c_) can trigger excitotoxicity, mitochondrial dysfunction, and neuronal death—processes implicated in the onset and progression of epilepsy [3,4,5].

In neurons, Ca^2+^ homeostasis is tightly regulated through fluxes between the cytosol and internal stores, primarily the endoplasmic reticulum (ER). Two families of Ca^2+^-release channels coordinate these movements: the inositol 1,4,5-trisphosphate receptor (InsP_3_R) and the ryanodine receptor (RyR) [6,7]. InsP_3_R-mediated Ca^2+^ release plays a central role in the generation of intracellular Ca^2+^ waves, which influence synaptic activity, dendritic integration, and gene transcription [8]. Perturbations in InsP_3_R and RyR function have been linked to epileptogenesis and neuronal hyperexcitability [9]. Despite this, the potential modulation of intracellular Ca^2+^ stores by antiepileptic drugs remains insufficiently explored.

Valproic acid (VPA) is one of the most widely prescribed broad-spectrum antiepileptic drugs, effective against generalized and focal seizures, as well as in bipolar disorder and migraine prophylaxis. Its primary mechanisms of action have traditionally been associated with the potentiation of γ-aminobutyric acid (GABA) neurotransmission [10,11], the modulation of glutamate uptake [12], and the inhibition of voltage-dependent sodium and T-type Ca^2+^ channels [13,14,15,16,17]. Additional studies have also suggested neuroprotective roles for VPA in reducing ER stress and lipid accumulation [18,19,20,21]. Yet, despite over six decades of clinical use, the precise molecular targets that mediate its anticonvulsant and neuroregulatory properties remain incompletely defined.

The ER is increasingly recognized as a key modulator of neuronal Ca^2+^ signaling and excitability [6]. Among the intracellular signaling systems regulating Ca^2+^ dynamics, the inositol 1,4,5-trisphosphate receptor (IP_3_R), located on the membrane of the endoplasmic reticulum (ER), plays a pivotal role in mobilizing Ca^2+^ from internal stores to the cytosol and nucleus [6,22,23]. The ER constitutes the major intracellular Ca^2+^ reservoir, maintaining distinct subcompartments of high and low Ca^2+^ concentrations that are tightly regulated to ensure proper signaling and neuronal viability [24,25,26].

The activation of IP_3_Rs by phospholipase C-derived IP_3_ results in finely tuned Ca^2+^ release events, which contribute to the generation of local and global Ca^2+^ waves that control neurotransmitter exocytosis and synaptic efficiency [27,28,29]. Moreover, the existence of different IP_3_R isoforms with tissue-specific distribution and functional diversity further refines this regulation, allowing neurons to adapt their Ca^2+^ signaling patterns to physiological and pathological stimuli [30,31].

Dysregulation of IP_3_-mediated Ca^2+^ release has been implicated in several neuropathological conditions, including excitotoxicity, ER stress, and epileptogenesis. Alterations in ER Ca^2+^ homeostasis can modify synaptic strength and neuronal excitability, thereby contributing to seizure propagation. Understanding how pharmacological agents, particularly antiepileptic drugs, interact with IP_3_R-dependent Ca^2+^ release pathways is therefore crucial for elucidating their cellular mechanisms of action and for developing novel therapeutic strategies targeting Ca^2+^ signaling in the nervous system.

Research using genetically encoded aequorin targeted to the ER has demonstrated that VPA induces Ca^2+^ release from InsP_3_-sensitive stores in a concentration-dependent manner, with an IC_50_ of approximately 17 µM, and kinetics strikingly similar to the second messenger InsP_3_ [22,23,24,25]. Pharmacological blockade of InsP_3_R with heparin or 2-aminoethyl diphenylborinate (2-APB) abolishes this effect, supporting the hypothesis that VPA induces InsP_3_R-mediated Ca^2+^ mobilization from the endoplasmic reticulum. Moreover, studies in PC12 neuronal-like cells and bovine chromaffin cells revealed that VPA enhances catecholamine release and modulates ER Ca^2+^ signaling without affecting RyR-mediated pathways [24,25,26,27,28,29]. These intracellular actions suggest that VPA can directly influence synaptic release machinery through modulation of ER Ca^2+^ dynamics.

Nevertheless, the relationship between VPA’s intracellular effects and its clinical efficacy remains controversial. While therapeutic concentrations of VPA suppress epileptiform activity in some models [14], paradoxical pro-epileptic effects have been reported in others [30,31,32], potentially reflecting patient-dependent variations in InsP_3_R sensitivity or downstream Ca^2+^ signaling. This duality may contribute to phenomena such as pharmacoresistance or idiosyncratic adverse responses in certain epileptic individuals.

Taken together, these findings highlight the need to further elucidate the intracellular mechanisms underlying VPA’s actions. The present study was designed to investigate how VPA modulates Ca^2+^ homeostasis at the level of the ER, focusing on its interaction with InsP_3_R and subsequent effects on neurotransmitter release. Understanding these mechanisms could provide a unifying framework linking VPA’s antiepileptic efficacy, its side-effect profile, and the cellular basis of variable patient responses. As far as we know, VPA has not been found yet to be implicated directly in the regulation of Ca^2+^ homeostasis at the ER level. We have therefore approached such a study here. Our findings suggest that VPA caused the release of Ca^2+^ from intracellular stores through a mechanism reminding of InsP_3_.

## 2. Results

### 2.1. Valproic Acid Induces Ca^2+^ Release from the Endoplasmic Reticulum

**Figure 1:**
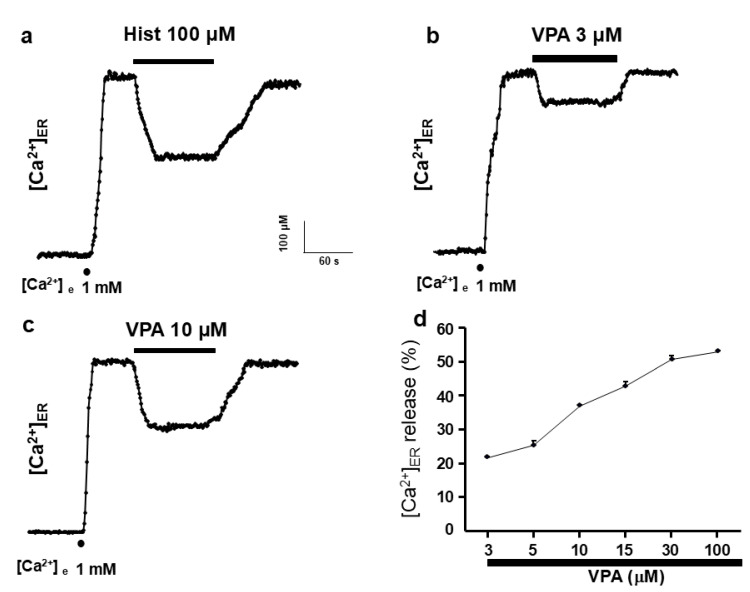
Valproic acid induces Ca^2+^ release from the endoplasmic reticulum in HeLa cells. (**a**), shows the effects of histamine (100 μM) on [Ca^2+^]_ER_. This original trace was obtained in 23 experiments from 5 different batches of cells. Plots (**b**,**c**) illustrate VPA action at 3 and 10 μM, respectively, on [Ca^2+^]_ER_ in intact HeLa cells. Once the ER was refilled with 1 mM of Ca^2+^ (as shown by dots), drugs were applied as shown in the horizontal bars at the top of the figure. VPA experiments are representative of 25, 20, and 28 experiments of each type from 6, 5, and 7 different cell batches, respectively. Plot (**d**) shows a concentration–response curve for VPA. The average percentage of Ca^2+^ released from the ER induced by increased concentrations of VPA 3, 5, 10, 15, 30, and 100 μM. Data are means ± s.e. Means from 25, 12, 20, 12, 28, and 25 different experiments from 6, 3, 5, 3, 7, and 6 cell batches, respectively.

After aequorin reconstitution with coelenterazine in ER-depleted conditions, the experiments were initiated by perfusing HeLa cells with a medium containing 1 mM Ca^2+^ to allow ER refilling (Figure 1a). Complete ER refilling required 30–60 s, reaching a steady-state [Ca^2+^]_ER_ of 570.53 ± 17.52 µM. When histamine (100 µM) was applied for 2 min, the expected Ca^2+^ release from the ER occurred, decreasing [Ca^2+^]_ER_ from approximately 600 µM to 400 µM (Figure 1a). Application of VPA (3 µM) produced a similar effect, reducing [Ca^2+^]_ER_ from 600 to 470 µM (Figure 1b), corresponding to a 37.36 ± 0.01% decrease. This effect was reversible. The concentration–response curve (Figure 1d) revealed a threshold at 3 µM and a maximal effect between 30 and 100 µM (approximately 55% ER release). The calculated IC_50_ value was approximately 17 µM.

### 2.2. ER Ca^2+^ Release Elicited by VPA in Permeabilized HeLa Cells

**Figure 2:**
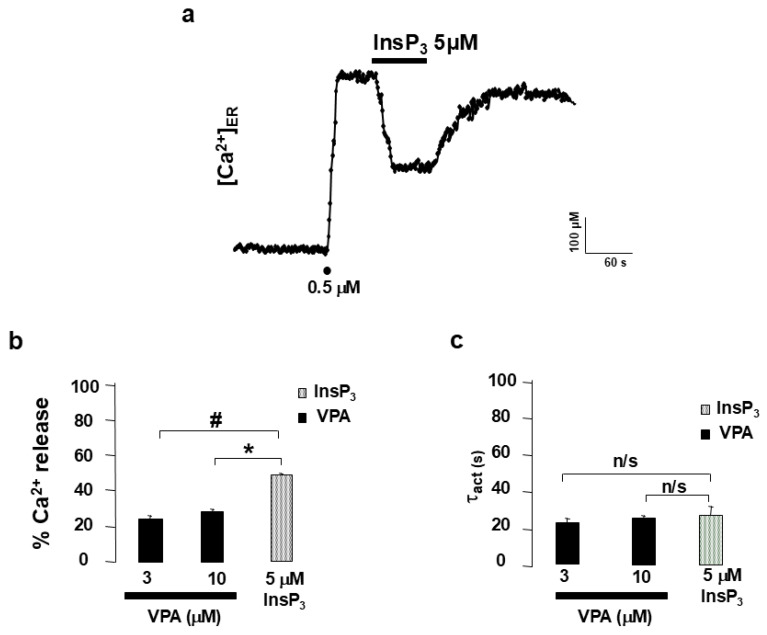
[Ca^2+^]_ER_ in permeabilized HeLa cells. Plot (**a**) shows the original traces of InsP_3_ 5 μM. After permeabilization, ER was refilled with 0.5 μM of Ca^2+^, as shown by dots. The drug was applied as indicated in the horizontal bars at the top of the figure. Plot (**b**) shows the original traces of VPA 10 μM. After permeabilization, the ER was refilled with 0.5 μM Ca^2+^, as shown by dots. The drug was applied as indicated in the horizontal bars at the top of the figure. Plot (**c**) shows average results expressed as τ_act_ for InsP_3_ and increasing concentrations of VPA application. Thus, 3 and 10 μM of VPA demonstrated similar kinetics to InsP_3_. Pooled data are means ± s.e. mean of 12 and 15 experiments from 3 and 4 different cell batches (VPA 3 and 10 μM, respectively). Means ± s.e. mean of InsP_3_ came from 20 experiments from 5 different cell batches. # indicates significant differences between InsP_3_ and VPA 3 μM. Asterisk (*) indicates significant differences between InsP_3_ and VPA 10 μM. n/s indicates not significant differences. An ANOVA test was performed.

To determine whether VPA acted via plasma membrane receptors or directly on intracellular targets, experiments were performed on digitonin-permeabilized HeLa cells expressing erAEQ. Following permeabilization with 100 µM digitonin, cells were superfused with intracellular buffer (IB) to obtain a stable baseline, after which ER refilling was achieved using 0.5 µM Ca^2+^. Application of InsP_3_ (5 µM) triggered a decrease in [Ca^2+^]_ER_ from 600 µM to 300 µM (Figure 2a). Averaged results (Figure 2b) showed that InsP_3_ and VPA released 49.26 ± 0.01% and ~25% of ER Ca^2+^, respectively. The activation time constants (τ_act_) for VPA- and InsP_3_-elicited Ca^2+^ release were comparable (Figure 2c), indicating similar kinetics.

### 2.3. Comparison of the Kinetics of ER Ca^2+^ Release Induced by CPA and VPA

**Figure 3:**
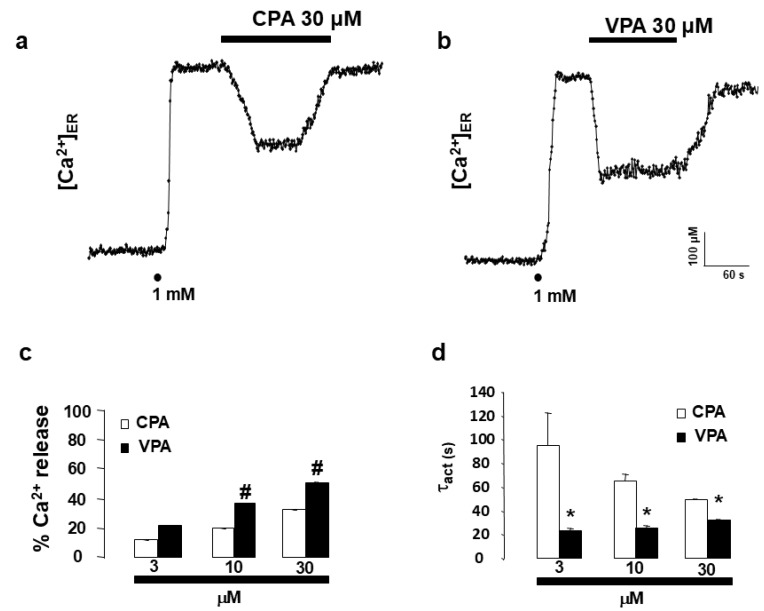
VPA follows a different mechanism from CPA. Original traces of CPA (30 μM, (**a**), a SERCA blocker, show different kinetics to VPA (30 μM, (**b**) in intact HeLa cells. Once the ER was refilled with 1 mM Ca^2+^ (as shown by dots), drugs were applied as shown in the horizontal bars at the top of the figure. Plot (**c**) represents average data expressed as % of Ca^2+^ release from ER, CPA, and VPA application. Plot (**d**) shows average results expressed as τ_act_ at increasing concentrations of VPA and CPA applications. For CPA 3, 10, and 30 μM pooled data are means ± s.e. mean of 10, 10, and 30 experiments from 3, 3, and 7 different cell batches; for VPA 3, 10, and 30 μM pooled data are means ± s.e. mean of 25, 20, and 28 experiments from 6, 5, and 7 different cell batches. Hash (#) indicates significant differences between VPA and CPA in terms of Ca^2+^ release. Asterisk (*) indicates significant differences between VPA and CPA when the τ_act_ is analyzed. An ANOVA test was performed.

To further understand the mechanism of VPA-induced ER Ca^2+^ release, its effect was compared with that of cyclopiazonic acid (CPA), a well-known inhibitor of the sarco/endoplasmic reticulum Ca^2+^-ATPase (SERCA). CPA (30 µM) decreased [Ca^2+^]_ER_ from 530 µM to 330 µM, corresponding to a 32.94 ± 0.10% release (Figure 3c). Lower concentrations of CPA (3 and 10 µM) elicited 11.74 ± 0.13% and 20.11 ± 0.15% Ca^2+^ release, respectively. In parallel experiments, VPA (3, 10, and 30 µM) produced 21.43 ± 0.10%, 37.36 ± 0.10%, and 51.10 ± 0.67% ER Ca^2+^ depletion, respectively (Figure 3b,c). Notably, VPA caused significantly faster ER Ca^2+^ release compared to CPA. The activation time constants (τ_act_) for CPA were 94.41 ± 28.63 s (3 µM), 64.97 ± 2.89 s (10 µM), and 49.15 ± 1.08 s (30 µM), whereas for VPA they were 23.18 ± 2.12 s (3 µM), 25.66 ± 1.83 s (10 µM), and 32.06 ± 1.06 s (30 µM) (Figure 3d).

### 2.4. [Ca^2+^]_ER_ Is Unaffected by 2-APB and Heparin

**Figure 4:**
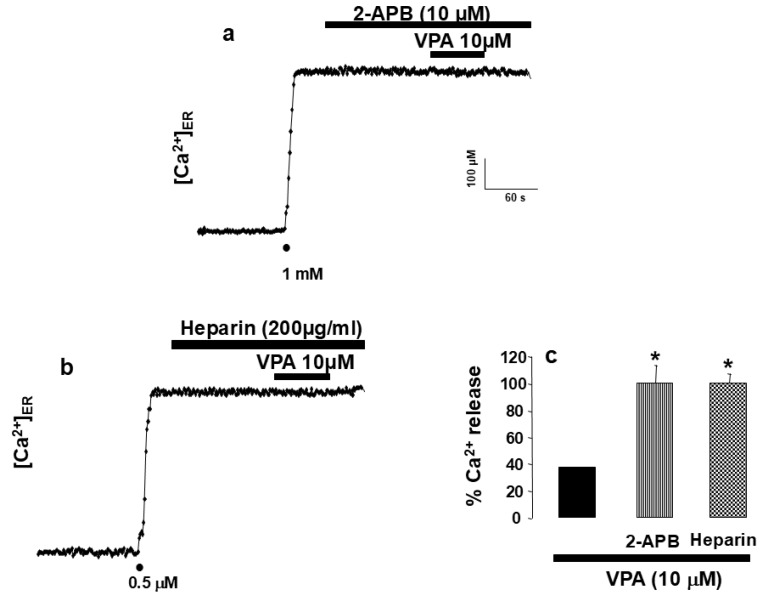
Inhibition of InsP_3_ receptors abolishes VPA-induced Ca^2+^ release from the ER. Plot (**a**) shows HeLa cells perfused with 2-APB at 10 μM before the addition of VPA 10 μM. Plot (**b**) shows HeLa cells treated with VPA (10 μM) and heparin (200 μg/mL). Drugs were applied as indicated in the horizontal bars at the top of the figure. Plot (**c**) shows pooled data from 12 and 15 experiments from 3 and 4 different cell batches. Asterisk (*) indicates significant differences between 2-APB and heparin with respect to VPA. An ANOVA test was performed.

To directly assess the involvement of the InsP_3_ receptor (InsP_3_R) in VPA-induced ER Ca^2+^ release, the InsP_3_R inhibitors 2-aminoethyl diphenylborinate (2-APB) and heparin were used. Pre-incubation with 10 µM 2-APB for 2 min abolished the VPA (10 µM)-induced ER Ca^2+^ release (Figure 4a). Similarly, in permeabilized HeLa cells, superfusion with 200 µg/mL heparin for 2 min before VPA application prevented any detectable change in [Ca^2+^]_ER_ (Figure 4b). The inhibitory effects of both agents are summarized in Figure 4c, confirming that VPA acts through InsP_3_-sensitive Ca^2+^ stores.

### 2.5. VPA Releases Ca^2+^ via InsP_3_R in PC12 Cells

**Figure 5:**
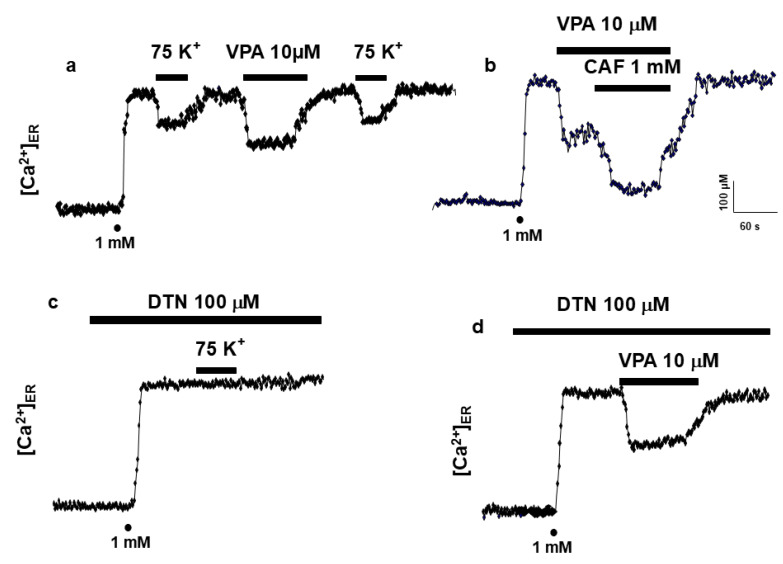
VPA stimulates release of Ca^2+^ from the ER in PC12 cells. Original traces of high K^+^ (75 mM) and VPA (10 μM, (**a**)), the coapplication of CAF (1 mM) and VPA (10 μM) showed different Ca^2+^ release from the ER to (**b**)) in intact PC12 cells. Once the ER was refilled with 1 mM Ca^2+^ (as shown by dots), drugs were applied as shown in the horizontal bars at the top of the figure. The perfusion of DTN (100 μM) abolished Ca^2+^ release from the ER induced by high K^+^ (75 mM) (**c**) but not VPA Ca^2+^ release (**d**). Pooled data are means ± s.e. mean of 10, from different cell batches (**a**); pooled data are means ± s.e. mean of 3 experiments from 2 different cell batches (**b**); pooled data are means ± s.e. mean of 9 experiments from 3 different cell batches (**c**,**d**).

To examine the specificity of VPA action on InsP_3_R, experiments were performed in PC12 cells, which express both InsP_3_R and RyR, as well as voltage-dependent Ca^2+^ channels (VDCCs) of the L- and N-type. Activation of VDCCs by depolarization with high K^+^ induces Ca^2+^ release through RyR. In ER-depleted PC12 cells expressing erAEQ, reintroduction of 1 mM Ca^2+^ restored [Ca^2+^]_ER_ (Figure 5a). Subsequent perfusion with high K^+^ or VPA (10 µM) elicited ER Ca^2+^ release via RyR or InsP_3_R, respectively. Co-application of caffeine (CAF) and VPA further enhanced ER Ca^2+^ release (Figure 5b). Dantrolene (DTN) (100 µM), an RyR inhibitor, abolished Ca^2+^ release triggered by K^+^ depolarization (Figure 5c) but not that induced by VPA (Figure 5d), confirming that VPA acts selectively on InsP_3_R-mediated pathways.

### 2.6. VPA Mitigates Intracellular Ca^2+^ Oscillations Induced by Veratridine

**Figure 6:**
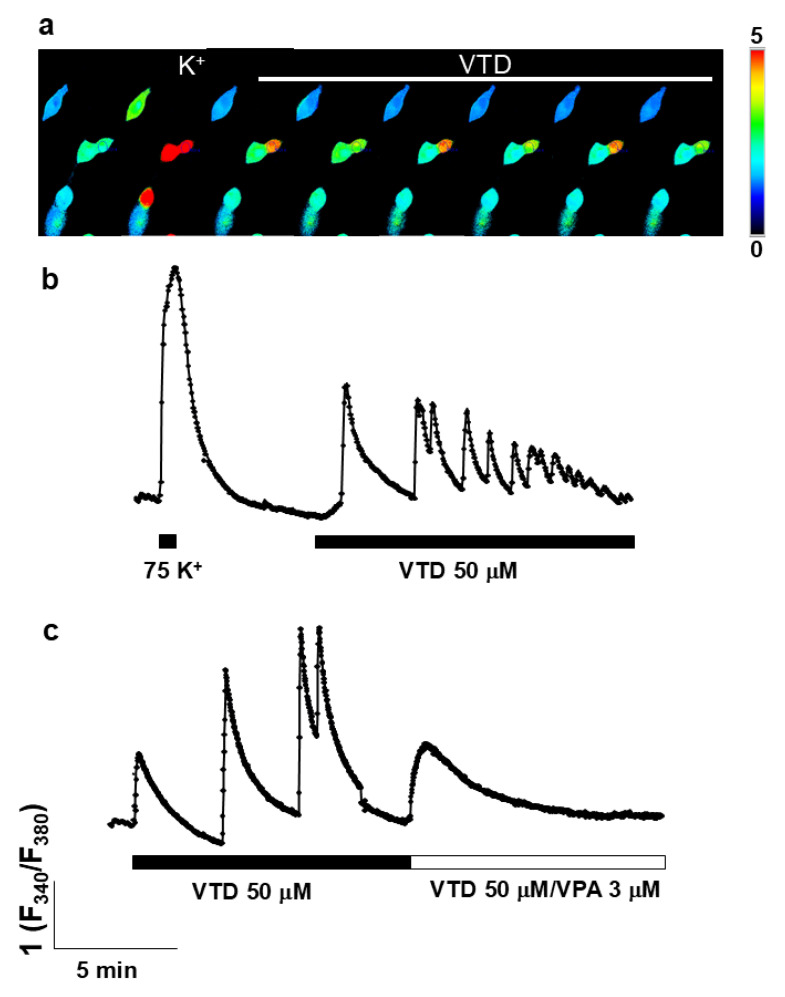
The effect of VPA on VTD epilepsy model in fura-2 loaded BCC. Panel a shows a row of images with the region of interest (roi) of [Ca^2+^]_c_ peak caused upon high K^+^ (75 mM) depolarization, followed by Ca^2+^ oscillations produced by VTD (as shown in horizontal bar) in the control solution. The pseudocolor scale indicates the increase in [Ca^2+^]_c_. Original traces in the absence (**b**) and in the presence of VPA (**c**) using the protocol described in (**a**). Horizontal bars at the bottom of the figure represent the application of the compounds. Experiments are representative of 3 to 6 of each type.

Given VPA’s clinical use in epilepsy, we investigated whether its ER Ca^2^-releasing effect modulates veratridine (VTD)-induced cytosolic Ca^2+^ oscillations, a cellular model of epileptiform activity. Application of VTD (50 µM) evoked rhythmic Ca^2+^ oscillations in fura-2-loaded bovine chromaffin cells (BCCs) (Figure 6a,b). Superfusion with VPA (30 µM) abolished these oscillations (Figure 6c) while simultaneously increasing basal [Ca^2+^]_c_. Thus, VPA suppresses VTD-induced Ca^2+^ oscillations, likely by releasing Ca^2+^ from ER stores.

### 2.7. VPA Facilitates Catecholamine Release in Bovine Chromaffin Cells

**Figure 7:**
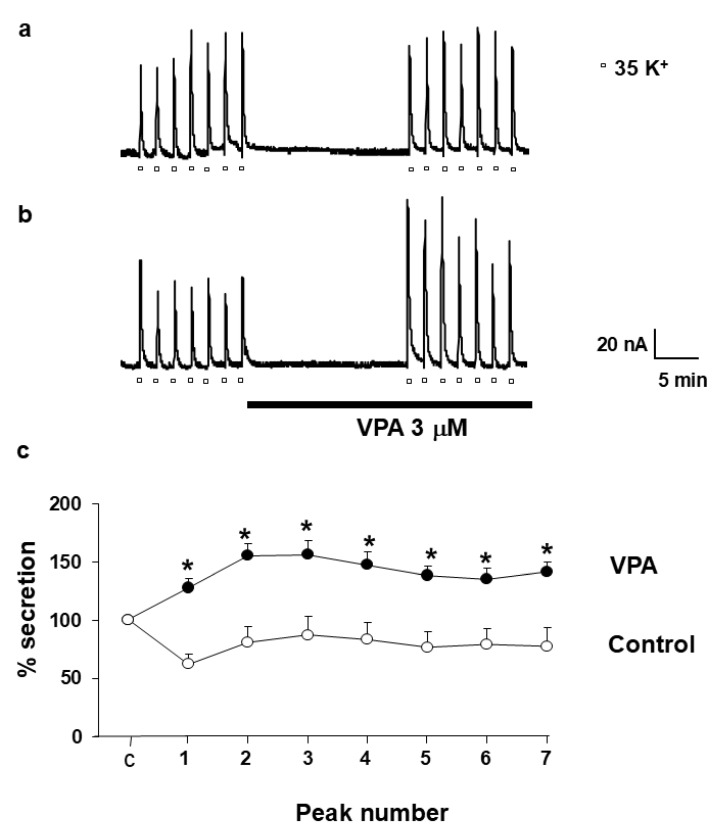
VPA potentiates the release of catecholamines in BCC. Plot (**a**) shows original traces of catecholamine secretory peaks induced by K^+^ (35 mM, during 5 s every 2 min) pulse in control cells. Plot (**b**) displays the enhanced response in the presence of VPA, as shown in the horizontal bar at the bottom of the figure. White circles represent control conditions, whereas black circles represent VPA-treated cells. Plot (**c**) illustrates the averaged results expressed as % of catecholamine secretion. Pooled data are means ± s.e. mean of 8 experiments from 3 different cell cultures. Asterisk (*) indicates significant differences in VPA with respect to control. An ANOVA test was performed.

To evaluate the functional consequences of VPA-induced ER Ca^2+^ release on neurotransmitter exocytosis, catecholamine secretion was measured in fast-superfused BCCs. Depolarization with 35 mM K^+^ for 5 s every 2 min evoked reproducible catecholamine release peaks averaging 80–90 nA (Figure 7a). Following 15 min of Krebs–Hepes superfusion, the mean peak value was 122.19 ± 4.31 nA. In the presence of VPA (3 µM), the average peak amplitude increased significantly to 198.20 ± 4.81 nA (Figure 7b). Data from eight independent experiments confirmed that VPA potentiated K^+^-evoked catecholamine release (Figure 7c), suggesting that ER Ca^2+^ mobilization by VPA enhances vesicular exocytosis.

## 3. Discussion

“The major finding of the present study is that the antiepileptic drug valproic acid (VPA) induces InsP_3_R-mediated Ca^2+^ release from the endoplasmic reticulum.” This conclusion is supported by the observation that, in HeLa cells, VPA induced a concentration-dependent Ca^2+^ release from the endoplasmic reticulum (ER) (Figure 1d). Importantly, the effect was not mediated by plasma membrane receptors, as it persisted in digitonin-permeabilized cells (Figure 2). In the absence of second messengers such as InsP_3_, VPA evoked Ca^2+^ release with kinetics remarkably similar to those triggered by InsP_3_ itself. The activation time constant (τ_act_) was almost identical for both compounds (Figure 2c), “indicating a functional convergence on InsP_3_R-dependent Ca^2+^ release mechanisms. In contrast, the kinetics of ER Ca^2+^ release elicited by VPA and by cyclopiazonic acid (CPA)—a specific inhibitor of the sarco/endoplasmic reticulum Ca^2+^-ATPase (SERCA)—were clearly different. These data indicate that VPA does not act through SERCA inhibition and that its effect depends on InsP_3_R-mediated ER Ca^2+^ mobilization.” This interpretation is further reinforced by the observation that the specific blockade of InsP_3_R using either 2-APB [27] or heparin [28] completely abolished VPA-induced ER Ca^2+^ release.

We acknowledge that both 2-aminoethoxydiphenyl borate (2-APB) and heparin exhibit complex pharmacological profiles and have been reported to display off-target effects on Ca^2+^ signaling [6]. However, their use at low concentrations has been extensively validated as a functional approach to assess InsP_3_ receptor (InsP_3_R) involvement in intracellular Ca^2+^ release pathways [6,8]. Importantly, heparin experiments were performed in digitonin-permeabilized cells, a classical strategy that minimizes indirect effects mediated by plasma membrane receptors or cytosolic signaling pathways and is widely accepted for functional interrogation of InsP_3_R-dependent Ca^2+^ release [25,29]. Moreover, despite their distinct chemical nature and mechanisms of action, both heparin and 2-APB fully abolished valproic acid–induced ER Ca^2+^ release, providing convergent pharmacological evidence for InsP_3_R involvement.

VPA specifically targets InsP_3_R but not RyR. The InsP_3_R appears to be the principal intracellular target of VPA. Although HeLa cells express ryanodine receptors (RyR), they do not contribute significantly to Ca^2+^ signaling [7]. To confirm the specificity of VPA for InsP_3_R, experiments were extended to PC12 cells, which express both InsP_3_R and RyR [21,22]. In these cells, K^+^ stimulation and VPA each triggered ER Ca^2+^ release in a two-step sequence. However, the caffeine-induced Ca^2+^ release via RyR was not affected by VPA, indicating that VPA selectively targets InsP_3_R-dependent Ca^2+^ stores.

VPA suppresses veratridine-induced Ca^2+^ oscillations. A particularly intriguing finding was that VPA abolished the cytosolic Ca^2+^ oscillations induced by veratridine (VTD) in chromaffin cells (Figure 6c). Because VTD is used as a cellular model of epileptiform activity [33,34,35,36], the ability of VPA to suppress these oscillations may have direct pathophysiological relevance. Interestingly, VPA itself increased cytosolic [Ca^2+^] (Figure 6c, right), likely reflecting InsP_3_R-mediated ER Ca^2+^ release. Such modulation could influence vesicular trafficking and neurotransmitter release, contributing to the drug’s antiepileptic effects. Previous studies have demonstrated that VPA can inhibit epileptiform discharges induced by VTD [34], yet paradoxically, VPA has also been reported to exert pro-epileptic effects under certain conditions [33]. Clinical reports describe cases of valproate-induced status epilepticus [10]. These divergent effects may depend on individual sensitivity of InsP_3_R subtypes to VPA, offering a potential explanation for both pharmacoresistance and proconvulsive reactions in some patients. Regarding the translational relevance of the concentrations of valproic acid (VPA) used in this study (3–30 µM), it is important to consider that VPA is highly bound to plasma proteins, mainly albumin, such that only a small fraction circulates as free drug [16]. Consequently, total plasma concentrations commonly used for therapeutic drug monitoring do not directly reflect the pharmacologically active fraction. Previous clinical and experimental studies have shown that VPA-induced intracellular Ca^2+^ signaling and secretory effects occur at low micromolar concentrations compatible with the free fraction of the drug [2,12,19]. Moreover, available pharmacological evidence indicates that brain and cerebrospinal fluid exposure to VPA is more closely related to unbound plasma concentrations than to total serum levels [16]. In this context, the concentration range of VPA effective in our experiments (3–30 µM) falls within the lower-to-mid range of clinically relevant free VPA concentrations. Finally, given that our study focuses on an intracellular target—the endoplasmic reticulum—additional intracellular compartmentalization or accumulation cannot be excluded, further supporting the physiological relevance of the concentrations used.

To our knowledge, this is the first comprehensive study demonstrating that VPA directly triggers Ca^2+^ release from the ER through InsP_3_R activation and enhances exocytosis. Although other authors have shown that VPA modulates insulin secretion [17], the mechanism underlying this effect was not linked to ER Ca^2+^ dynamics. Similarly, Yamamoto et al. (1997) [30] reported that chronic VPA exposure upregulated sodium channels and increased catecholamine secretion in adrenal chromaffin cells, but without considering ER Ca^2+^ homeostasis.

Furthermore, several studies have explored the role of VPA in bipolar disorder by analyzing its effects on Ca^2+^ signaling pathways [2,15,19]. Akimoto and colleagues found that VPA inhibited serotonin-induced Ca^2+^ responses in human platelets in a concentration-dependent manner, possibly involving protein kinase C (PKC) modulation. However, none of these investigations examined whether VPA acts directly on ER Ca^2+^ stores, as revealed in our present study.

We propose that VPA-induced Ca^2+^ release from the ER via InsP_3_R may represent an effective mechanism to regulate secretion and synaptic transmission. If similar phenomena occur in neurons, VPA-triggered ER Ca^2+^ mobilization could contribute to synaptic plasticity and neurotransmitter modulation. Under pathological conditions such as epilepsy, this mechanism might help attenuate the propagation of epileptic discharges by enhancing GABA release, thereby counteracting neuronal hyperexcitability [6,16,17,18,19,20,21,22,23,24,25,26,27,28,29,30,31,32,33,34,35,36,37,38,39,40,41,42].

## 4. Materials and Methods

### 4.1. HeLa Cell Culture and Transfection

HeLa cells were cultured in Dulbecco’s Modified Eagle’s Medium (DMEM) supplemented with 10% fetal calf serum (FCS). For transfection, cells were seeded onto 13 mm glass coverslips and grown to 60–70% confluence. Transfection was performed using 4 µg of plasmid DNA encoding the genetically engineered photoprotein aequorin. The mutated aequorin with low Ca^2+^ affinity targeted to the endoplasmic reticulum (erAEQ) was employed, as described previously [37]. Transfection was achieved using the Ca^2+^ phosphate method [38]. Experiments aimed at measuring changes in endoplasmic reticulum Ca^2+^ concentration ([Ca^2+^]_ER_) were performed 36 h post-transfection.

### 4.2. PC12 Cell Culture and Transfection

PC12 cells were maintained in DMEM supplemented with 7.5% fetal calf serum, 7.5% horse serum, 2 mM glutamine, 25 U/mL penicillin, and 25 µg/mL streptomycin. Cells were seeded on 13 mm poly-L-lysine-coated glass coverslips in 24-well plates and allowed to reach 60–70% confluence after 24 h at 37 °C in a humidified 5% CO_2_ atmosphere. Transfection with the erAEQ plasmid was achieved using Metafectene (Biontex Laboratories, Munich, Germany) [39]. Measurements of [Ca^2+^]_ER_ were performed 36–48 h after transfection.

### 4.3. Bovine Adrenal Chromaffin Cell Culture

Bovine chromaffin cells (BCCs) were isolated according to standard procedures with minor [40]. Cells were suspended in DMEM containing 5% FCS, 50 IU/mL penicillin, and 50 µg/mL streptomycin. For secretion experiments, 5 × 10^6^ cells were plated in 5 cm Petri dishes and maintained at 37 °C in a 5% CO_2_/95% air atmosphere. Cells were used between 1 and 5 days after plating.

### 4.4. Measurement of [Ca^2+^]_ER_ Changes with Aequorin

Two experimental conditions were used:

(a) Intact cells: The monolayer was superfused with Krebs–Hepes buffer for HeLa (KHBH) containing (in mM) 125 NaCl, 5 KCl, 1 Na_3_PO_4_, 1 MgSO_4_, 5.5 glucose, and 20 HEPES, pH 7.4, at room temperature (24 ± 2 °C), supplemented with 1 mM CaCl_2_.
(b) Permeabilized cells: An intracellular-like buffer (IB) was used containing (in mM) 140 KCl, 10 NaCl, 1 K_3_PO_4_, 10 HEPES, 1 MgCl_2_, 1 ATP, 5 succinate, and 20 µM ADP, pH 7.0, supplemented with 0.5 µM CaCl_2_.

Reconstitution of ER-targeted aequorin was achieved by incubating cells for 1–2 h in KHBH or KHBPC12 supplemented with 5 µM coelenterazine n, 5 µM ionomycin, and 600 µM EGTA. After loading, cells were washed with buffer containing 2% bovine serum albumin (BSA) and 1 mM EGTA. During experiments, 1 mM CaCl_2_, histamine, VPA, CPA, DTN, caffeine, and 2-APB were added as indicated in figure legends. Permeabilization was performed using 100 µM digitonin for 30 s. IB containing 0 Ca^2+^/100 µM EGTA was applied until stabilization, followed by IB containing 0.5 µM Ca^2+^. Luminescence was measured using a purpose-built luminometer. Calibration to [Ca^2+^] was achieved by adding excess Ca^2+^ (10 mM) in KHBH or KHBPC12 supplemented with 100 µM digitonin to expose the aequorin to maximal Ca^2+^.

### 4.5. On-Line Measurement of Catecholamine Release

Cells were gently detached using a rubber policeman and centrifuged at 800 rpm for 10 min. The pellet was resuspended in Krebs–Hepes buffer containing (in mM): 144 NaCl, 5.9 KCl, 1.2 MgCl_2_, 11 glucose, 10 HEPES, and 1 Ca^2+^ (pH 7.4). The suspension was placed in a jacketed microchamber superfused at 2 mL/min at room temperature. Catecholamine secretion was continuously monitored “on-line” by an electrochemical detector (Metrohm AG CH-9100, Herisau, Switzerland) operating in amperometric mode [41]. Secretion was evoked by 5 s pulses of high K^+^ (75 mM) every 2 min.

### 4.6. Single-Cell [Ca^2+^]c Measurements

Single-cell [Ca^2+^]c was determined at room temperature in fura-2-loaded cells as described previously [42]. Excitation wavelengths were alternated between 340 and 380 nm, and emitted light at 520 nm was collected and analyzed using CellR^®^ software version 2.8). Data were expressed as the fluorescence ratio F340/F380.

### 4.7. Chemicals

Coelenterazine n was obtained from Labnet Biotecnica (Madrid, Spain). CPA, histamine, InsP_3_, VPA, veratridine (VTD), dantrolene (DTN), 2-APB, caffeine, and heparin were purchased from Sigma-Aldrich (Madrid, Spain). Fura-2 was from Molecular Probes. The cDNA encoding ER-targeted aequorin was a generous gift from Prof. Tullio Pozzan. Reagens. Dulbecco’s Modified Eagle’s Medium (DMEM), fetal calf serum (FCS, and antibiotics were purchased from Gibco, Thermo Fisher Scientific, Waltham, MA, USA. Fura-2: Molecular Probes, Eugene, OR, USA. Poly-L-lysine-coated, CPA, histamine, InsP_3_, VPA, veratridine, dantrolene, 2-APB, caffeine, heparin: Sigma-Aldrich, St. Louis, MO, USA.

### 4.8. Statistics

Values are expressed as mean ± SE. Statistical significance was determined using one-way ANOVA. Differences were considered significant at *p* < 0.05.

A summary of reagent concentrations and literature justification is provided in Table S1.

## 5. Conclusions

In conclusion, our results reveal a novel intracellular mechanism of action for the classical antiepileptic drug VPA. “The drug induces InsP_3_R-dependent Ca^2+^ release from endoplasmic reticulum stores.” Considering the central role of InsP_3_-dependent Ca^2+^ signaling in neuronal excitability and epileptogenesis, this mechanism offers promising insights into the pharmacological action of VPA. “Although direct ligand–receptor binding was not assessed, the strict functional dependence on InsP_3_R activity demonstrated by kinetic, pharmacological, and organellar Ca^2+^ measurements supports InsP_3_R-mediated Ca^2+^ mobilization as an intracellular effect of valproic acid.” Future studies should focus on identifying the molecular determinants of VPA–InsP_3_R interaction and their implications for antiepileptic resistance and neuronal Ca^2+^ homeostasis in chronic epilepsy.

## Data Availability

The data presented in this study are openly available in Preprints.org, section Medicine and Pharmacology, at https://doi.org/10.20944/preprints202512.1137.v1.

## Supplementary Materials

The following supporting information can be downloaded at: https://www.mdpi.com/article/10.3390/ijms27031176/s1.
