# Supplementary material for: Valproic Acid Stimulates Release of Ca2+ from InsP3-Sensitive Ca2+ Stores
Source: Int J Mol Sci. 2026 Jan 23;27(3):1176. doi: 10.3390/ijms27031176 (PMC12898086; doi:10.3390/ijms27031176)
Supplement: Supplementary file 1 [file ijms-27-01176-s001.zip › ijms-4075595-supplementary.pdf]

Supplementary Table S1. Reagents, concentrations, and literature justification.

Reagent	Concentration(s) used	Experimental rationale	Key references
Valproic acid (VPA)	3–100 µM	Low micromolar range selected to investigate direct intracellular effects on Ca^{2+} signaling, close to reported therapeutic plasma concentrations. Similar concentrations have been widely used in neuronal and neuroendocrine Ca^{2+} studies.	Löscher, 2002, CNS Drugs; Yamamoto et al., 1997, J Neurochem; Kurita et al., 2007, Prog Neuropsychopharmacol Biol Psychiatry
Inositol 1,4,5-trisphosphate ($InsP_3$)	5 µM	Standard concentration in permeabilized-cell preparations to induce robust but non-saturating ER Ca^{2+} release, allowing kinetic comparisons with drug-induced responses.	Montero et al., 1997, J Cell Biol; Pizzo et al., 1997, J Cell Biol; Alvarez & Montero, 1999, Cell Calcium
2-Aminoethyl diphenylborinate (2-APB)	10 µM	Established concentration for effective inhibition of $InsP_3$ receptor–mediated Ca^{2+} release without major effects on RyR-dependent pathways or ER Ca^{2+} loading.	Ehrlich et al., 1994, Trends Pharmacol Sci; Zima et al., 2007, J Physiol

Heparin	200 µg/mL	Widely used intracellular antagonist of $InsP_3$ receptors in permeabilized-cell systems.	Montero et al., 1997, J Cell Biol; Ehrlich et al., 1994, Trends Pharmacol Sci
Cyclopiazonic acid (CPA)	3–30 µM	Classical SERCA inhibitor used to induce dose-dependent ER Ca^{2+} depletion and to compare kinetics with non-SERCA mechanisms.	Pizzo et al., 1997, J Cell Biol; Alvarez & Montero, 1999, Cell Calcium
Digitonin	100 µM	Selective permeabilization of the plasma membrane while preserving ER integrity; standard concentration in aequorin-based ER Ca^{2+} measurements.	Montero et al., 1997, J Cell Biol; Alvarez & Montero, 1999, Cell Calcium
Extracellular Ca^{2+} (intact cells)	1 mM	Physiological Ca^{2+} concentration allowing controlled ER refilling without Ca^{2+} overload.	Rizzuto, 2001, Curr Opin Neurobiol
Intracellular Ca^{2+} (permeabilized cells)	0.5 µM	Enables controlled ER refilling and reproducible $InsP_3R$ activation in permeabilized preparations.	Pizzo et al., 1997, J Cell Biol
High K^+	35–75 mM	Standard depolarizing stimulus to activate voltage-	García-Sancho et al., 1999, J Cell Biol; Cano-Abad et al., 1998, Br J

		dependent Ca^{2+} channels and Ca^{2+}-dependent secretion.	Pharmacol; Cano-Abad et al., J Chem Biol, 2001
Caffeine	1 mM	Classical ryanodine receptor activator used to distinguish RyR- from $InsP_3R$-dependent ER Ca^{2+} release.	Fasolato et al., 1991, J Biol Chem; García-Sancho et al., 1999, J Cell Biol
Dantrolene	100 µM	Selective ryanodine receptor inhibitor used to confirm $InsP_3R$ specificity of VPA-induced Ca^{2+} release.	Mori et al., 2005, Epilepsy Res
Veratridine	50 µM	Established concentration to induce sustained Na^+ channel activation and epileptiform Ca^{2+} oscillations.	Otoom & Alkadhi, 1998, Brain Res; Otoom & Alkadhi, 2000, Epilepsy Res
